# The correlation between serum total bile acid and adverse perinatal outcomes in pregnant women with intrahepatic cholestasis of pregnancy (ICP) and non-ICP hypercholanemia of pregnancy

**DOI:** 10.1080/07853890.2024.2331059

**Published:** 2024-03-21

**Authors:** Peizhen Zhang, Zhangmin Tan, Chuo Li, Zhenyan Han, Jin Zhou, Yuzhu Yin

**Affiliations:** Department of Obstetrics and Gynecology, The Third Affiliated Hospital of Sun Yat-Sen University, Guangzhou, China

**Keywords:** Total bile acid, adverse perinatal outcomes, liver disease, asymptomatic hypercholanemia of pregnancy, intrahepatic cholestasis in pregnancy

## Abstract

**Background:**

The association between excessive serum total bile acid (TBA) and adverse perinatal outcomes in individuals with non-intrahepatic cholestasis of pregnancy (non-ICP) hypercholanemia has not been determined, and it is unclear if this link is similar to that observed in patients with ICP.

**Objective:**

To examine the adverse perinatal outcomes in two specific subcategories: those with ICP and those with non-ICP, including individuals with liver disease and asymptomatic hypercholanemia of pregnancy (AHP), at different levels of TBA. Investigate the correlation between TBA levels and adverse perinatal outcomes of ICP, liver disease, and AHP.

**Methods:**

From 2013 to 2021, pregnant women with excessive TBA levels were taken from the electronic medical record database of our hospital and categorized into three groups: ICP (*n* = 160), liver disease (*n* = 164), and AHP (*n* = 650). This was done as part of a retrospective cohort research project. Multivariable regression and subgroup analyses were performed to examine the association between TBA levels and adverse perinatal outcomes in each group.

**Results:**

The study found no significant differences in adverse perinatal outcomes between the ICP and liver disease groups at different TBA levels. However, at moderate TBA levels, both groups had a higher risk of adverse perinatal outcomes than the AHP group (*p* < 0.017). Among liver disease cases with TBA ≥ 100µmol/L, three cases of perinatal deaths (6.67%) associated with moderate-to-severe acute hepatitis occurred between 27 and 33 weeks of gestation. A 59% higher chance of perinatal death was found for every 10 µmol/L rise in TBA, even after significant variables and confounders were taken into account (adjusted odds ratio (aOR) = 1.59; 95% confidence interval (CI): 1.06–2.40; *p* = 0.03).

**Conclusions:**

If a pregnant woman has moderate-to-severe liver disease and TBA ≥ 100µmol/L, preterm termination of pregnancy (before 34 weeks) may be considered.

## KEY MESSAGES

If someone doesn’t have ICP but does have moderate-to-severe hepatitis and TBA levels of 100 µmol/L or more, they should be treated more aggressively, and their pregnancies should be terminated earlier (before 34 weeks) than what is usually done for ICP.

## Introduction

In intrahepatic cholestasis of pregnancy (ICP), having excessive serum total bile acid (TBA) has been linked to a higher risk of adverse perinatal outcomes [1–3]. Over the years, various countries have issued guidelines for the diagnosis and management of ICP to mitigate adverse perinatal outcomes caused by TBA [4,5]. However, there are also cases of pregnancy-related non-ICP hypercholanemia, and no consensus has been reached on the optimal management of these cases.

Liver disease and asymptomatic hypercholanemia of pregnancy (AHP) serve as the main exclusion criteria for diagnosing ICP [4–6] and represent the predominant non-ICP hypercholanemia population during pregnancy. It is unclear if the association between TBA and adverse perinatal outcomes in individuals with non-ICP hypercholanemia (including liver disease and AHP) is similar to that observed in patients with ICP.

The term liver disease with hypercholanemia refers to a group of disorders characterized by hypercholanemia and varying degrees of hepatocellular injury (elevated ALT). Hepatitis B virus (HBV) infection, a risk factor for adverse foetal outcomes, has been associated with both ICP and liver disorders during pregnancy in China [7–9]. A meta-analysis from 2020 demonstrated that pregnant women with HBV infection had a higher risk of developing ICP, and ICP patients, in turn, had an increased risk of HBV infection [10]. Interestingly, it was found that the combination of ICP with HBV carriage without hepatocellular injury did not increase the risk of adverse pregnancy outcomes compared to ICP alone [11]. Therefore, it remains unclear whether liver disease with hepatocellular injury at the same TBA level elevates the risk of adverse perinatal outcomes compared to ICP.

AHP is a recently recognized clinical condition characterized by hypercholanemia during pregnancy. Initially, it was considered a sub-health state not associated with adverse perinatal outcomes [12–14]. On the contrary, recent studies suggest that AHP may be associated with higher rates of adverse perinatal outcomes than ICP [15,16]. However, according to a cohort study, although the risks of adverse foetal outcomes in AHP (28.3%) are higher than those in normal pregnancies (8.9%), they are still lower than those in the ICP [17]. Consequently, the contribution of AHP to adverse perinatal outcomes remains controversial.

In this study, we hypothesized that the incidence of adverse pregnancy outcomes is consistent in different groups of ICP, AHP, and liver disease at the different TBA levels. The primary objective of this retrospective study was to compare adverse perinatal outcomes among patients with ICP, liver disease, and AHP at the different TBA levels. The secondary objective was to explore the correlation of TBA with adverse pregnancy outcomes in patients with hypercholanemia during pregnancy, including further subgroup analyses in the ICP, liver disease, and AHP groups.

## Materials and methods

### Study design and study population

In this retrospective cohort study, data were retrieved from an electronic medical record database from January 2013 to December 2021 at the Third Affiliated Hospital of Sun Yat-Sen University in Guangdong Province, China. The data extracted encompassed maternal demographic characteristics, medical and obstetric histories, and maternal and infant outcomes. Two obstetricians from our team identified eligible women and collected relevant patient data over a three-month period. The Ethics Committee of the Third Affiliated Hospital of Sun Yat-sen University reviewed and approved the study in accordance with the Declaration of Helsinki’s principles (reference number: [2021] 02-400-01). Given that the data were anonymized, the requirement for informed consent was waived.

All pregnant women with TBA levels ≥10 µmol/L (hypercholanemia), including fasting and postprandial TBA, were identified using laboratory computer systems. Pregnant women were included when their TBA levels were ≥10 µmol/L, with or without signs of pruritus during pregnancy, as documented in the hospital records. Pregnancies complicated by severe congenital malformations, chromosomal abnormalities, and/or multiple congenital anomalies and those delivered before 24 weeks or at other hospitals were excluded from the study. Patients were stratified into three categories: the ICP group, the liver disease group, and the AHP group (non-ICP hypercholanemia of pregnancy included the liver disease group and the AHP group). Those with pruritus and hypercholanemia not due to liver disease were classified as the ICP group, those with hypercholanemia due to liver disease as the liver disease group, and those without pruritus and with hypercholanemia not due to liver disease as the AHP group [5,17–19].

TBA levels were measured at least twice for each patient during the entire pregnancy (from 11–14 weeks to delivery). All cases were actively managed and treated with ursodeoxycholic acid (UDCA) until delivery, according to the Chinese clinical guidelines [5]. The highest measured TBA value throughout pregnancy was used for analysis, consistent with the literature [20–22].

The primary outcome of interest was adverse perinatal outcomes, including preterm birth, early preterm birth, low Apgar score, meconium staining of the amniotic fluid, neonatal unit admission, and perinatal death. From the hospital records of all the cases that were studied, information was gathered on the mother’s age, whether she was a first-time mother or a primigravida, her level of education, her obstetric and medical history, biochemical parameters (including aspartate aminotransferase [AST], alanine aminotransferase [ALT], and γ-glutamyl transpeptidase [GGT]), and any bad outcomes during pregnancy.

### Sample size

The number of subjects required for this study was estimated, as previously described in the literature [23]. It is well-established that TBA is associated with adverse outcomes in patients with ICP, including preterm delivery (29%), meconium-stained amniotic fluid (15%), a low Apgar score (2%), neonatal unit admission (20%), and perinatal death (1%). The prevalence of neonatal unit admissions was found to be 20%. For sample size determination, a two-sided test with a confidence level of 95% and an error margin of 0.03% was utilized, leading to a minimum requirement of 685 patients [24]. Considering an additional 20% for contingencies, this study must have at least 856 patients.

### Statistical analysis

Patient characteristics were evaluated across three groups based on the causes of maternal hypercholanemia: the ICP group, the liver disease group, and the AHP group. ALT, TB, and GGT levels were measured when the peak TBA levels were observed. Data were expressed as the mean and standard deviation for normally distributed variables or as the median and interquartile range (IQR) for skewed variables. Differences among groups were analyzed using a one-way ANOVA. Qualitative data were described as percentages and analyzed using the chi-square (χ2) or Fisher’s exact test. Missing data were imputed using multiple imputations. Multivariate logistic regression analyses were performed to assess the association between TBA levels and perinatal outcomes. TBA levels were treated as a continuous variable, with odds ratios calculated per 10 μmol/L TBA increase. The analyses were initially conducted using a crude model. Further analyses included cumulative adjustments for maternal age, primigravida, primiparous, twin pregnancies, abnormal hepatic ultrasound, autoimmune disease, and history of hypercholanemia [4,21,25]. Subgroup analyses were performed using logistic regression and presented in a forest plot. Statistical analyses were conducted using the statistical software packages R 3.3.2 (http://www.R-project.org, R Foundation) and the Free Statistics software platform (Beijing, China) [26]. A two-sided *p*-value <0.05 was statistically significant, and a two-sided *p*-value <0.0176 (0.05/3) indicated statistical significance for subgroup analysis.

### Definitions

Hypercholanemia in pregnant women was diagnosed based on excessive TBA levels (≥10 µmol/L), including fasting and postprandial bile acids. TBA levels were measured on at least two occasions per patient throughout the diagnosis and stratification process to improve diagnostic and stratification accuracy [20]. In our study, liver disease was identified when high levels of liver transaminases and TBA were seen. These levels could be caused by a number of diseases, such as hepatitis A, B, C, D, and E viruses, immune system damage to the liver, and other things. Pruritus, predominantly on the palms and soles, during the second or third trimester was associated with elevated serum TBA levels (≥10 µmol/L) [19]. Hypercholanemia was categorized into three groups based on TBA levels: mild hypercholanemia (TBA ≥10 µmol/L and TBA <40 µmol/L), moderate hypercholanemia (TBA ≥40 µmol/L and TBA <100 µmol/L), and severe hypercholanemia (TBA ≥100 µmol/L) [19,20]. A history of hypercholanemia was considered positive if the disease had been present in any previous pregnancy in that particular case. A history of hepatobiliary disease was defined as hepatitis A, B, or C, a history of elevated liver transaminases with a different cause, or cholecystectomy for cholelithiasis. Following previous literature, subgroup analyses were done on three groups: those with pruritus and high hypercholanemia that wasn’t caused by liver disease (ICP group); those with high hypercholanemia that was caused by liver disease (liver disease group); and those without pruritus and high hypercholanemia that wasn’t caused by liver disease (AHP group). The analysis of adverse perinatal outcomes included preterm birth (defined as <37 weeks of gestation), early preterm birth (defined as <34 weeks of gestation), iatrogenic preterm labour due to TBA (early termination of pregnancy, including vaginal induction and caesarean section, due to excessive TBA concentration), caesarean section, NST/CST foetal distress (abnormal NST or CST), meconium staining of the amniotic fluid, spontaneous preterm birth, low Apgar score (≤7 before 5 min), neonatology department admission, and perinatal death (intrauterine foetal death from 24 weeks of gestation to delivery and death of perinatal infants). For twin pregnancies, perinatal outcome statistics were based on the foetus with the most severe adverse outcome.

## Results

### Demographics of participants

**Figure 1.**
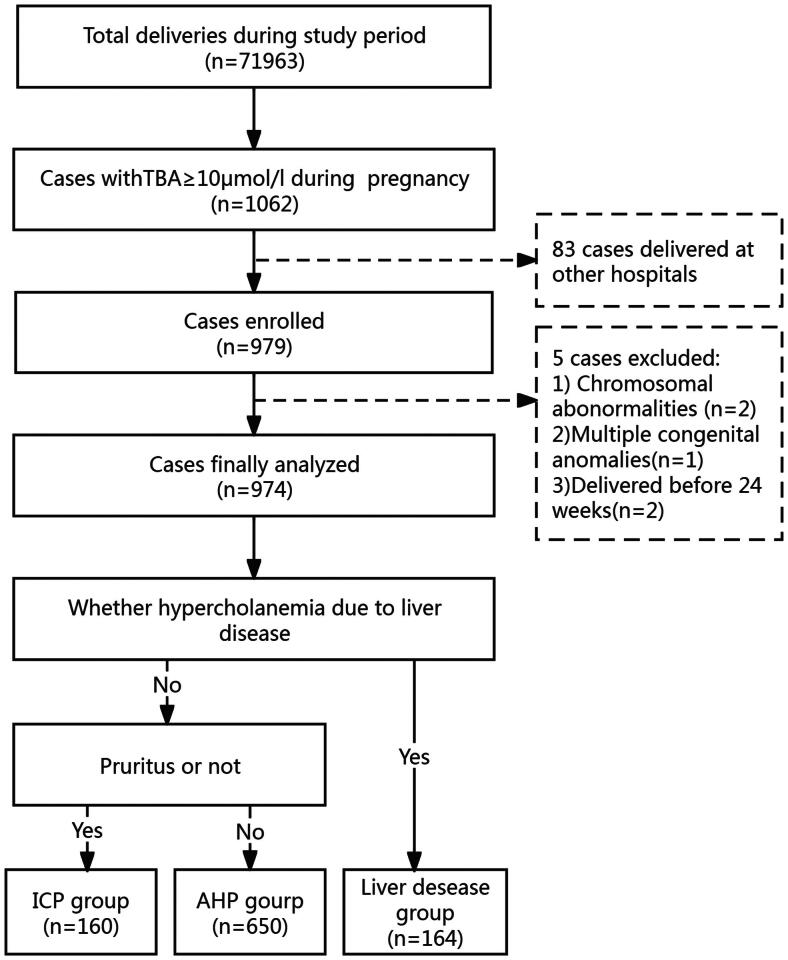
Flowchart of the Study Population. TBA: total bile acid; ICP: intrahepatic cholestasis of pregnancy; AHP: asymptomatic hypercholanemia of pregnancy.

During the 9-year study period (2013–2021), a total of 71,963 pregnant women gave birth, and 1062 cases with TBA ≥10 µmol/L were assessed. Cases with limited data or delivery at another hospital (*n* = 83) and combined severe malformations (*n* = 5) were excluded. Finally, 974 women were analyzed, and the perinatal outcomes were retrieved from the hospital records of these cases. Participants were divided into three groups based on aetiology: the ICP group (*n* = 160, 16.43%), the liver disease group (*n* = 164, 16.84%), and the AHP group (*n* = 650, 66.74%) (Figure 1). See the flowchart in Figure 1. Table 1 presents a comparison of the groups’ demographic and clinical characteristics, as well as the distribution of TBA levels.

**Table 1.** Maternal demographics within the study population.

Baseline demographic	Total population	ICP	Non-ICP		*p* value
			Liver disease	AHP	
	(*n* = 974)	(*n* = 160)	(*n* = 164)	(*n* = 650)	
Maternal age	30.28 ± 4.71	29.29 ± 4.63	30.15 ± 4.97	30.56 ± 4.63	< 0.01
Middle school degree or less	155 (15.91)	31 (19.37)	28 (17.07)	96 (14.77)	0.42
Primigravida	372 (38.19)	71 (44.38)	56 (34.15)	245 (37.69)	0.15
Multigravida	602 (61.81)	89 (55.62)	108 (65.85)	405 (62.31)	
Primiparous	586 (60.16)	103 (64.38)	100 (60.98)	383 (58.92)	0.44
Multiparous	388 (39.84)	57 (35.62)	64 (39.02)	267 (41.08)	
Hepatitis virus carrier	284(29.16)	47(29.38)	93(56.71)	144(22.15)	< 0.01
Hepatitis B virus carrier	275 (28.23)	46 (28.75)	87 (53.05)	142 (21.85)	
Hepatitis C virus carrier	7 (0.72)	1 (0.62)	5 (3.05)	1 (0.15)	
Hepatitis E virus carrier	1 (0.10)	0 (0)	1 (0.61)	0 (0)	
Hepatitis A virus carrier	1 (0.10)	0 (0)	0 (0)	1 (0.15)	
Gallstones	22 (2.26)	5 (3.12)	5 (3.05)	12 (1.85)	0.42
Cholecystitis	95 (9.75)	26 (16.25)	29 (17.68)	40 (6.15)	< 0.01
Cholecystic polypus	66 (6.78)	12 (7.50)	15 (9.15)	39 (6)	0.33
Fatty liver	118 (12.11)	17 (10.62)	38 (23.17)	63 (9.69)	< 0.01
Cirrhosis	8 (0.82)	3 (1.88)	4 (2.44)	1 (0.15)	< 0.01
Abnormal hepatobiliary ultrasound (simple)^a^	141 (14.48)	32 (20)	24 (14.63)	85 (13.08)	0.08
Autoimmune diseases	9 (0.92)	0 (0)	4 (2.44)	5 (0.77)	0.06
History of hyperbileacidemia	66 (6.78)	6 (3.75)	6 (3.66)	54 (8.31)	0.03
Hypercholanemia (special)^b^	208 (21.36)	44 (27.50)	164 (100)	0 (0)	< 0.01
Pruritus	160 (16.43)	160 (100)	0 (0)	0 (0)	< 0.01
Twin pregnancies	53(5.44)	11 (6.88)	13(7.93)	29(4.46)	0.15
In-vitro fertilization	49(5.03)	9(5.62)	11(6.71)	29(4.46)	0.47
Hypertensive disorders of pregnancy	60(6.16)	1(0.62)	18(10.98)	41(6.31)	< 0.01
GDM or PGDM	167(17.15)	23(14.40)	32(19.51)	112(17.23)	0.57
AST^c^	21.00	52.50	77.50	18.00	< 0.01
	(16.00, 45.50)	(20.00, 152.50)	(39.20, 192.50)	(15.00, 23.00)	
ALT^d^	16.00	83.00	120.50	12.00	< 0.01
	(11.00, 54.20)	(13.20, 243.00)	(46.00, 316.00)	(9.20, 18.00)	
GGT^e^	12.00	25.00	32.00	9.00	< 0.01
	(8.00, 29.50)	(12.00, 49.80)	(21.00, 53.50)	(7.00, 14.00)	
TBA	41.90	60.00	67.90	33.80	< 0.01
	(26.30, 62.30)	(38.70, 90.20)	(50.00, 105.70)	(22.70, 50.30)	
10 µmol/L ≤ TBA < 40 µmol/L (mild hypercholanemia)	462 (47.43)	44 (27.50)	19 (11.59)	399 (61.38)	< 0.01
40 µmol/L ≤ TBA < 100 µmol/L (moderate hypercholanemia)	410 (42.09)	82 (51.25)	100 (60.98)	228 (35.08)	< 0.01
TBA ≥ 100 µmol/L (severe hypercholanemia)	102 (10.47)	34 (21.25)	45 (27.44)	23 (3.54)	< 0.01

ICP: intrahepatic cholestasis of pregnancy; AHP: asymptomatic hypercholanemia of pregnancy; GDM: gestational diabetes mellitus; PGDM: pregestational diabetes mellitus; AST: aspartate aminotransferase; ALT: alanine aminotransferase; GGT: γ-glutamyl transpeptidase; TBA: serum total bile acid.

Continuous variables are shown as mean ± SD, or median interquartile range.

Categorical variables are presented as numbers (percentages).

*p* < 0.017 compared to ICP.

^a^ Hepatobiliary ultrasound abnormalities without clearly associated liver pathologies.

^b^ Hypercholanemia was combined with elevated liver enzymes and hepatic disease. In the liver disease group, hypercholanemia was caused by hepatic disease, while there was no clear correlation between hypercholanemia and hepatic disease in the other two groups.

^c^ Data is missing for 78 (8.00%) patients.

^d^ Data is missing for 144 (14.8%) patients.

^e^ Data is missing for 359 (36.9%) patients.

### The perinatal outcomes of the three subgroups at different TBA levels

After further stratification according to TBA levels, the perinatal outcomes of the three subgroups were compared. We found no significant differences in adverse perinatal outcomes between the ICP and liver disease groups, regardless of TBA levels. Additionally, there were no significant differences in adverse perinatal outcomes between the AHP and ICP groups at mild and severe TBA levels. However, at moderate TBA levels, the AHP group had significantly lower rates of preterm birth, caesarean section, meconium staining of amniotic fluid, and neonatology department admission compared to the ICP and liver disease groups (*p* < 0.017) (Table 2).

**Table 2.** Perinatal outcomes in pregnant women with ICP, liver disease, or AHP at different levels of TBA.

Perinatal outcomes	Study population					Mild hypercholanemia					Moderate hypercholanemia					Severe hypercholanemia				
	Total population	ICP	Non-ICP		*p* value	Total population	ICP	Non-ICP		*p* value	Total population	ICP	Non-ICP		*p* value	Total population	ICP	Non-ICP		*p* value
			Liver disease	AHP				Liver disease	AHP				Liver disease	AHP				Liver disease	AHP	
	(*n* = 974)	(*n* = 160)	(*n* = 164)	(*n* = 650)		(*n* = 462)	(*n* = 44)	(*n* = 19)	(*n* = 399)		(*n* = 410)	(*n* = 82)	(*n* = 100)	(*n* = 228)		(*n* = 102)	(*n* = 34)	(*n* = 45)	(*n* = 23)	
Gestational age at diagnosis, weeks	30.42 ± 8.17	30.96 ± 6.69	30.85 ± 8.15	30.17 ± 8.50	0.42	31.98 ± 7.86	32.53 ± 6.45	31.96 ± 8.78	31.93 ± 7.97	0.89	29.25 ± 8.18	30.32 ± 7.05	31.37 ± 8.25	27.93 ± 8.30	< 0.01	28.02 ± 8.23	30.49 ± 5.92	29.24 ± 7.60	21.97 ± 9.58	< 0.01
Gestational age at delivery^b^, weeks	37.17 ± 2.31	36.18 ± 2.29	36.12 ± 2.69	37.68 ± 2.03	< 0.01	38.10 ± 1.85	37.62 ± 1.60	37.44 ± 2.48	38.18 ± 1.83	0.05	36.83 ± 2.04	36.29 ± 1.84	36.73 ± 2.31	37.06 ± 1.96	0.01	34.39 ± 2.59	34.07 ± 2.50	34.22 ± 2.66	35.20 ± 2.53	0.23
Preterm birth^bf^	328 (33.68)	84 (52.50)	91 (55.49)	153 (23.54)	< 0.01	67 (14.50)	8 (18.18)	5 (26.32)	54 (13.53)	0.20	182 (44.39)	49 (59.76)	50 (50)	83 (36.40)	< 0.01	79 (77.45)	27 (79.41)	36 (80)	16 (69.57)	0.59
Full-term birth	646 (66.32)	76 (47.50)	73 (44.51)	497 (76.46)		395 (85.50)	36 (81.82)	14 (73.68)	345 (86.47)		228 (55.61)	33 (40.24)	50 (50)	145 (63.60)		23 (22.55)	7 (20.59)	9 (20)	7 (30.43)	
Early preterm birth^b^	95 (9.75)	29 (18.12)	36 (21.95)	30 (4.62)	< 0.01	15 (3.25)	2 (4.55)	2 (10.53)	11 (2.76)	0.11	30 (7.32)	10 (12.20)	8 (8)	12 (5.26)	0.11	50 (49.02)	17 (50)	26 (57.78)	7 (30.43)	0.10
Spontaneous preterm birth	34 (3.49)	6 (3.75)	5 (3.05)	23 (3.54)	0.94	13 (2.81)	1 (2.27)	1 (5.26)	11 (2.76)	0.51	16 (3.90)	3 (3.66)	2 (2)	11 (4.82)	0.57	8 (7.84)	2 (5.88)	4 (8.89)	2 (8.70)	0.90
Cesarean section^bf^	677 (69.51)	129 (80.62)	144 (87.80)	404 (62.15)	< 0.01	254 (54.98)	28 (63.64)	11 (57.89)	215 (53.88)	0.45	331 (80.73)	70 (85.37)	92 (92)	169 (74.12)	< 0.01	92 (90.20)	31 (91.18)	41 (91.11)	20 (86.96)	0.84
Vaginal birth	297 (30.49)	31 (19.38)	20 (12.20)	246 (37.85)		208 (45.02)	16 (36.36)	8 (42.11)	184 (46.12)		79 (19.27)	12 (14.63)	8 (8)	59 (25.88)		10 (9.80)	3 (8.82)	4 (8.89)	3 (13.04)	
Iatrogenic preterm labor because of TBA^bf^	228 (23.41)	69 (43.12)	71 (43.29)	88 (13.54)	< 0.01	16 (3.46)	3 (6.82)	2 (10.53)	11 (2.76)	0.05	145 (35.37)	42 (51.22)	39 (39)	64 (28.07)	< 0.01	67 (65.69)	24 (70.59)	30 (66.67)	13 (56.52)	0.54
NST fetal distress	62 (6.37)	5 (3.12)	6 (3.66)	51 (7.85)	0.03	49 (10.61)	3 (6.82)	3 (15.79)	43 (10.78)	0.54	10 (2.44)	1 (1.22)	2 (2)	7 (3.07)	0.75	3 (2.94)	1 (2.94)	1 (2.22)	1 (4.35)	1.00
Meconium-stained amniotic fluid^bf^	147 (15.09)	33 (20.62)	41 (25)	73 (11.23)	< 0.01	50 (10.82)	4 (9.09)	3 (15.79)	43 (10.78)	0.66	65 (15.85)	17 (20.73)	23 (23)	25 (10.96)	< 0.01	32 (31.37)	12 (35.29)	15 (33.33)	5 (21.74)	0.52
Low Apgar score^b^	32 (3.29)	5 (3.12)	12 (7.32)	15 (2.31)	< 0.01	15 (3.25)	1 (2.27)	3 (15.79)	11 (2.76)	0.02	9 (2.20)	2 (2.44)	3 (3)	4 (1.75)	0.67	8 (7.84)	2 (5.88)	6 (13.33)	0 (0)	0.18
Neonatology department admission^bf^	427 (43.84)	96 (60)	100 (60.98)	231 (35.54)	< 0.01	154 (33.33)	19 (43.18)	8 (42.11)	127 (31.83)	0.23	197 (48.05)	49 (59.76)	61 (61)	87 (38.16)	< 0.01	76 (74.51)	28 (82.35)	31 (68.89)	17 (73.91)	0.40
Rooming-in	547 (56.16)	64 (40)	64 (39.02)	419 (64.46)		308 (66.67)	25 (56.82)	11 (57.89)	272 (68.17)		213 (51.95)	33 (40.24)	39 (39)	141 (61.84)		26 (25.49)	6 (17.65)	14 (31.11)	6 (26.09)	
Perinatal death	4 (0.41)	0 (0)	3 (1.83)	1 (0.15)	0.04	0	0	0	0	–	1 (0.24)	0 (0)	0 (0)	1 (0.44)	–	3 (2.94)	0 (0)	3 (6.67)	0 (0)	0.34

ICP: intrahepatic cholestasis of pregnancy; AHP: asymptomatic hypercholanemia of pregnancy; CI: confidence interval; NST: non-stress test; TBA: serum total bile acid.

Continuous variables are shown as the mean ± SD.

Categorical variables are presented as numbers (percentages).

*p* < 0.017 compared to ICP.

^a^indicates statistically significant differences between the ICP and liver groups in the total study population.

^b^indicates statistically significant differences between the ICP and AHP groups in the total study population.

^c^indicates statistically significant differences between the ICP and liver groups in the study population with mild hypercholanemia.

^d^indicates statistically significant differences between the ICP and AHP groups in the study population with mild hypercholanemia.

^e^indicates statistically significant differences between the ICP and liver groups in the study population with moderate hypercholanemia.

^f^indicates statistically significant differences between the ICP and AHP groups in the study population with moderate hypercholanemia.

^g^indicates statistically significant differences between the ICP and liver groups in the study population with severe hypercholanemia.

^h^indicates statistically significant differences between the ICP and AHP groups in the study population with severe hyperchola.

In our study population, perinatal death occurred in 0.41% (*n* = 4/974) of the included patients. Notably, 6.67% (*n* = 3/45) of those with liver disease and severe hypercholanemia (TBA ≥ 100µmol/L) experienced perinatal death (*p* < 0.01). All three patients had moderate-to-severe acute hepatitis and were delivered between 27 and 33 weeks. In the group with moderate hypercholanemia, only 0.44% (*n* = 1/228) of perinatal deaths occurred after 37 weeks and were due to AHP.

### Correlation of TBA level and perinatal outcomes in three subgroups

**Figure 2.**
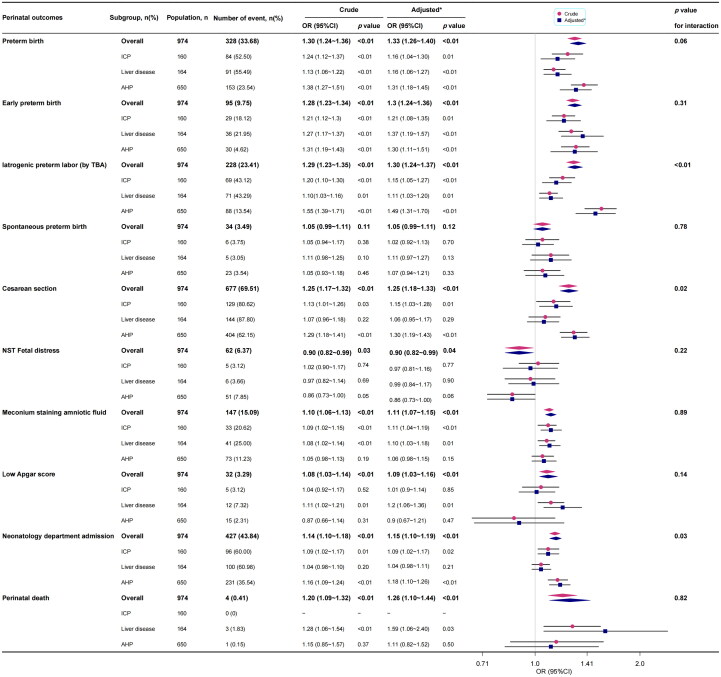
Forest plots of logistic regression analyses of adverse outcomes for the foetus for every 10 μmol/L rise in TBA of ICP, liver disease, and AHP. CI: confidence interval; ICP: intrahepatic cholestasis of pregnancy; TBA: serum total bile acid; AHP: asymptomatic hypercholanemia of pregnancy. *: adjustment for maternal age, primigravida, primiparous, twin pregnancies, abnormal hetpatic ultrasound, autoimmune disease, and history of hyperbileacidemia. Significance levels: *p* < 0.05.

TBA levels, as a measure of hypercholanemia, were strongly associated with adverse perinatal outcomes among the ICP, liver disease, and AHP groups, independent of important covariates and confounders. For every 10 μmol/L increase in TBA, the probability of preterm birth, early preterm birth, iatrogenic preterm labour, caesarean section, meconium-stained amniotic fluid, neonatology department admission, and perinatal death increased by 33% (adjusted odds ratio (aOR)=1.33; 95% confidence interval (CI): 1.26–1.4; *p <* 0.01), 30% (aOR = 1.30; 95%CI: 1.24–1.36; *p <* 0.01), 30% (aOR = 1.30; 95%CI: 1.24–1.37; *p <* 0.01), 25% (aOR = 1.25; 95%CI: 1.18–1.33; *p <* 0.01), 11% (aOR = 1.11; 95%CI: 1.07–1.15; *p <* 0.01), 15% (aOR = 1.15; 95%CI: 1.10–1.19; *p <* 0.01), and 26% (aOR = 1.26; 95%CI: 1.10–1.44; *p <* 0.01), respectively. However, there was no significant association between higher TBA levels and spontaneous preterm delivery (aOR = 1.05; 95% CI: 0.99–1.11; *p* = 0.12) (Figure 2).

Elevated TBA concentrations in the liver disease group significantly increased the risk of perinatal meconium-stained amniotic fluid and a low Apgar score, thus significantly increasing the risk of perinatal death. For every 10 μmol/L increase in TBA level, the risk of perinatal death increased by 59% (aOR, 1.59; 95%CI: 1.06–2.40). However, it was not the main cause of caesarean sections and neonatology department admissions (Figure 2).

For the AHP group, elevated TBA concentration was linked to iatrogenic preterm labour, caesarean section, and consequently, preterm birth, early preterm birth, and neonatology department admission. However, it was not linked to NST foetal distress, meconium staining of amniotic fluid, or a low Apgar score (Figure 2).

## Discussion

In this retrospective cohort study, we hypothesized that adverse pregnancy outcomes were similar in different groups of ICP, AHP, and liver disease at the same TBA levels and that the effect of TBA on adverse pregnancy outcomes would be consistent. We looked at the perinatal outcomes of the ICP, liver disease, and AHP groups and found that the liver disease and ICP groups had significantly worse perinatal outcomes at different TBA levels. However, the incidence rates of preterm birth, earlier preterm birth, caesarean section, meconium staining of amniotic fluid, and neonatology department admission in the AHP group were lower than in the other two groups. Compared to the ICP group, the liver disease group had a higher rate of perinatal deaths between 27 and 33 weeks of pregnancy. There was also a stronger link between perinatal death and TBA when TBA was 100 µmol/L or higher. Elevated TBA concentration was the primary reason for caesarean sections in the AHP group, which significantly increased the risk of neonatology department admission, preterm birth, and early preterm birth.

Several noteworthy clinical observations regarding liver disease with hypercholanemia warrant emphasis. Researchers have already looked at studies with hepatitis patients and found that HBsAg-positive ICP patients had much higher levels of serum TBA and a higher risk of bad outcomes during pregnancy [9]. However, these studies did not compare the risk of poor pregnancy outcomes at the same TBA levels between ICP and other hypercholanemias in pregnancy. Our results confirmed that severe hypercholanemia was more frequent in patients with liver disease and hepatitis. Additionally, we used TBA levels to identify differentially adverse perinatal outcomes in ICP compared to those in liver disease. As expected, no significant differences were found between the ICP and liver disease groups in adverse perinatal outcomes at different TBA levels, although the liver disease group was associated with a higher HBV infection rate than the ICP group. This discrepancy could be attributed to HBV-infected ICP patients without hepatitis being similar to ICP patients in terms of adverse perinatal outcomes [11] or TBA levels being the most important factor affecting adverse perinatal outcomes, regardless of the aetiology [27,28]. However, the analysis of perinatal death was limited due to the small case numbers.

In our study, perinatal death occurred in 0.41% (*n* = 4/974) of the included patients, of whom 6.67% (*n* = 3/45) had TBA ≥ 100µmol/L and moderate-to-severe acute hepatitis, occurring between 27 and 33 weeks of gestation. The risk of perinatal death in liver disease with severe hypercholanemia (6.67%) was significantly higher than reported in the Royal College of Obstetricians and Gynaecologists (RCOG) clinical guidelines for severe ICP (3.44%) [18]. This result was similar to some earlier research on ICP, which found that a peak TBA level of ≥100µmol/L was linked to a higher risk of perinatal death [19]. This could be because of foetal arrhythmia [27,29] or acute placental vessel spasm [30,31]. We also found that the risk of perinatal death increased by 59% for every 10 μmol/L increase in TBA level in liver disease. Liver diseases, including hepatitis virus carriage, non-alcoholic liver disease, cirrhosis, and immune liver disease, have been associated with adverse pregnancy outcomes such as preterm delivery, FGR, and perinatal death [25,32]. The presence of risk factors or co-morbidities can increase the risk of stillbirth in women with ICP, according to the ICP clinical guideline published in the British Journal of Obstetrics and Gynaecology (BJOG) in 2022 [18]. There is a lot of evidence that hepatitis increases oxidative stress during the development of hypercholanemia [33–35], which leads to higher levels of total TBA in the peripheral blood compared to ICP [9,30,36]. Therefore, in patients who have TBA ≥100µmol/L and also have liver disease, excessive TBA concentrations and liver disease could work together to cause oxidative stress, which raises the risk of perinatal death. For patients with TBA ≥100µmol/L and moderate to severe liver function abnormalities, more aggressive and earlier termination of pregnancy, even before 34 weeks of gestational age, should be considered to prevent perinatal death. However, for other hypercholanemias due to liver disease in pregnancy, clinicians can refer to ICP clinical management guidelines.

Furthermore, noteworthy clinical observations related to AHP warrant emphasis. Firstly, women with AHP had a more frequent history of hypercholanemia than ICP, indicating that hereditary factors might be involved in AHP pathogenesis. Additionally, AHP women (*n* = 142) were more likely to have HBV infection (21.8%) than women of childbearing age (2%–8%), which could be due to HBV infection being a high-risk factor for hypercholanemia in pregnancy. Previous studies on ICP have shown a higher risk of ICP among HBV-infected pregnant women and an increased risk of HBV infection among ICP patients [8,10]. This finding is consistent with our study, which found a significant difference in the prevalence of HBV infection between pregnant women with and without ICP. Similar observations were found in AHP. Therefore, HBV infection poses a higher risk not only for ICP but also for AHP.

At present, whether AHP causes adverse perinatal outcomes remains controversial [17]. Our study found a significantly higher risk of preterm birth and neonatology department admission in AHP than in normal pregnancies [37], although the risk was significantly lower than that in the ICP and liver disease groups. This difference could be attributed to the predominance of mild hypercholanemia in the AHP group, while moderate hypercholanemia was prevalent in the ICP and liver disease groups. We also discovered that AHP had a lower risk of bad outcomes than ICP and liver disease in people with moderate hypercholesterolaemia. This may be because AHP had lower TBA levels than the other two groups when they had moderate hypercholesterolaemia. Previous studies have reported that elevated TBA levels are associated with adverse outcomes, such as foetal cardiac arrhythmia and placental vessel spasms, consistent with our results [27,30,31,38]. Therefore, adverse perinatal outcomes are lower in the AHP group, where mild hypercholanemia is dominant, than in other hypercholanemias during pregnancy. However, TBA remains a valuable indicator of adverse outcomes in the perinatal period.

Although the past decade has witnessed unprecedented medical progress, the relationship between TBA and adverse perinatal outcomes in AHP has been largely underexplored. Initial literature on AHP considered it not to cause adverse perinatal outcomes [13,14], but later studies suggested that AHP can lead to perinatal death [15,16]. Our study found that 0.15% (*n* = 1/650) of stillbirths in the AHP group occurred after 37 weeks, similar to the incidence in America (0.1%–0.3%) [19]. A recent study from Chongqing reached the new conclusion that adverse perinatal outcomes were higher in AHP than in the general population and lower than in ICP [17]. However, this study did not quantify the effect of TBA concentration on adverse perinatal outcomes in the AHP population. In our study, excessive TBA levels were an important factor in caesarean delivery, preterm delivery, and subsequent admission to the neonatal department in the AHP population. However, they did not result in foetal distress and neonatal asphyxia, as shown by abnormal neonatal NST, low Apgar scores, or abnormal amniotic fluid. When dealing with hypercholanemia pregnancies, doctors tend to prematurely terminate the pregnancy to avoid potential consequences, as previous studies on ICP suggested. Putting together our study findings with those from previous studies, ending the pregnancy early because of too much worry about how TBA might affect the adverse delivery outcomes could be a major reason for early delivery and neonatal transfer in the AHP group, as seen in ICP [39]. However, the risks of preterm delivery for the newborn should be thought about [40]. Based on our experience, we do not recommend termination of pregnancy before 37 weeks, as TBA concentrations are not significantly associated with perinatal distress in cases of AHP.

The present study had some limitations that should be acknowledged. First, as it focused on Chinese pregnant women, the applicability of the results may be more relevant to the Chinese population, necessitating future multicentre studies to validate these findings across diverse populations. Second, our study’s sample size was insufficient to assess rare populations and outcomes, such as those with autoimmune liver disease, acute fatty liver during pregnancy, severe hepatitis, and perinatal birth. Finally, the assessment of perinatal outcomes was based on Chinese clinical guidelines, which might differ in stringency from other clinical guidelines.

In this cohort study, we discovered that if a woman has TBA levels of 100 µmol/L or more and moderate to severe liver dysfunction, it might be better to end the pregnancy more quickly and more aggressively (before 34 weeks) in order to save the life of the unborn child. However, premature termination of pregnancy (before 37 weeks) was not recommended in cases of AHP, whose TBA concentrations are not significantly associated with perinatal distress.

## Data Availability

Upon reasonable request, the corresponding author will provide the dataset.
